# Retrospective cohort study of epitope-based virtual crossmatch allocation in a public transplant system (Pernambuco, Brazil)

**DOI:** 10.3389/fimmu.2026.1879817

**Published:** 2026-07-28

**Authors:** Rafael Melo Santos de Serpa Brandão, Adalberto Socorro da Silva, Luís Gustavo Modelli de Andrade, Luiz Claudio Demes da Mata Sousa, João Marcelo Medeiros de Andrade, Frederico Castelo Branco Cavalcanti, Glauco Henrique Willcox, Samuel de Alencar Cavalcante, Ruy de Lima Cavalcanti Neto, Amaro Medeiros de Andrade, Alexandre de Holanda Cavalcanti Pinto, Diogo Buarque Cordeiro Cabral, Tatiana Cristina Manzi Sena dos Santos, Isa Maria Teixeira Leão, Elizabeth Lima Guimarães, Bruno de Melo Correa, André Bezerra Pereira do Rego, Semiramis Jamil Hadad do Monte

**Affiliations:** 1 Laboratório de Imunogenética e Biologia Molecular (LIB-UFPI), Universidade Federal do Piauí, Teresina, Brazil; 2 Departamento de Biologia, Universidade Federal do Piauí, Teresina, Brazil; 3 Departamento de Medicina Interna, Universidade Estadual Paulista Júlio Mesquita Filho (UNESP), Botucatu, Brazil; 4 Unidade Geral de Transplante, Instituto de Medicina Integral Professor Fernando Figueira (IMIP), Recife, Brazil; 5 Unidade de Nefrologia, Real Hospital Português de Beneficência em Pernambuco, Recife, Brazil; 6 Laboratório HLA Diagnóstico, Recife, Brazil; 7 Central de Transplantes de Pernambuco, Secretaria de Saúde do Estado de Pernambuco, Recife, Brazil

**Keywords:** EpViX, highly sensitized patients, kidney transplantation, prioritization, virtual crossmatch

## Abstract

Highly sensitized kidney transplant candidates face major barriers to transplantation because broad anti-HLA antibody reactivity substantially limits donor compatibility. We conducted a retrospective cohort study comparing two allocation eras in Pernambuco, Brazil: a pre-EpViX era (2005–2014; n=3,136) and an EpViX era (2016–2025; n=4,544). We evaluated the association between an epitope-informed definition of unacceptable antigens integrated with virtual crossmatching and access to deceased-donor kidney transplantation. The proportion of candidates who underwent transplantation was higher during the EpViX era (50%) than during the pre-EpViX era (36%) (p<0.001), while median waiting time among transplanted recipients decreased from 22 to 7 months (p<0.001). Among highly sensitized candidates (cPRA ≥80%), the estimated probability of transplantation at 12 months was 17% in the EpViX era compared with 3% in the pre-EpViX era. In adjusted analyses, listing during the EpViX era was associated with a higher likelihood of transplantation (HR 2.55; 95% CI 2.37–2.74; p<0.001). Epitope-level virtual crossmatch demonstrated high concordance with prospective complement-dependent cytotoxicity crossmatch, with sensitivity of 98.7%, specificity of 93.4%, and a negative predictive value of 99.6%. An allocation strategy incorporating an epitope-informed definition of unacceptable antigens was associated with greater access to deceased-donor kidney transplantation, including among highly sensitized candidates, while maintaining high concordance with prospective crossmatch testing.

## Introduction

Highly sensitized kidney transplant candidates, characterized by broad anti-human leukocyte antigen (HLA) antibody repertoires and commonly quantified using calculated panel reactive antibody (cPRA) or virtual PRA (vPRA), continue to face substantial barriers to transplantation. In practical terms, their immunologic profile excludes a large fraction of otherwise available donors, which translates into prolonged waiting times and reduced access to transplantation compared with non-sensitized individuals (1–3). This disadvantage is not confined to a single system. Across different allocation frameworks, similar patterns emerge, although with varying magnitude. In fact, data from the United Kingdom indicate that sensitized candidates may remain on the waiting list for extended periods, with median times approaching 74 months (4). In Germany, candidates with vPRA levels above 85% show a markedly reduced likelihood of transplantation, even within systems designed to account for sensitization (2). In the United States, despite significant allocation reforms, individuals with cPRA values exceeding 99.9% remain among the most disadvantaged groups in terms of transplant access (5). These observations, taken together, suggest that conventional allocation strategies only partially address the underlying immunologic constraints.

Attempts to mitigate the above-mentioned disparities have relied on prioritization rules and acceptable mismatch programs. Some of these approaches, such as the Eurotransplant Acceptable Mismatch program, have improved access for selected groups of highly sensitized patients (2–6). Even so, their effectiveness is inherently limited by the use of antigen-level matching and by the difficulty of identifying compatible donors for individuals with rare HLA phenotypes. As a result, a proportion of highly sensitized candidates continues to experience prolonged waiting times and a persistently low probability of transplantation (2–6). Other factors - including blood group distribution, geographic constraints, and logistical aspects of allocation - further contribute to this imbalance (7).

At the core of this problem lies the way antibody reactivity is represented. Traditional allocation systems rely predominantly on antigen-level definitions, yet growing evidence indicates that antibody recognition is more accurately described at the level of epitopes (eplets). When interpreted at this level, reactivity patterns become more informative: it becomes possible to distinguish clinically meaningful antibody responses from those that are unlikely to result in adverse outcomes. From a practical standpoint, this enables a more precise and biologically grounded definition of unacceptable antigens, which can then be incorporated into allocation decisions (8).

Brazil offers a relevant setting to examine these concepts. The country operates a large, publicly funded transplant system coordinated through the National Transplant System (SNT), with a steady increase in kidney transplantation activity over recent decades (9–11). Within this structure, the state of Pernambuco introduced an allocation strategy that explicitly incorporates epitope-level information. The approach is based on the definition of unacceptable antigens informed by antibody reactivity patterns and integrates virtual crossmatching as a screening step prior to transplantation. The analytical framework supporting this strategy did not emerge in isolation. It builds on the epitope-based concepts introduced by HLAMatchmaker (12–14), which enabled the identification of antibody targets at a structural level but remained operationally demanding in routine practice. To address these limitations, our group developed EpHLA, an automated platform for epitope reactivity analysis that demonstrated high concordance with HLAMatchmaker while significantly reducing processing time (15, 16). Subsequently, the Epitope Virtual Crossmatch platform (EpViX) was developed to integrate these analytical capabilities with virtual crossmatch execution and allocation support (17, 18).

In October 2016, this framework was operationalized in Pernambuco through the Kidney Allocation with Prioritization and Negative Virtual Crossmatch program. The program embeds the definition of unacceptable antigens and the requirement for a negative virtual crossmatch into the allocation workflow, while remaining fully aligned with national regulatory priorities. Based on this background, we hypothesized that an allocation strategy grounded in epitope-informed definition of unacceptable antigens would improve access to deceased-donor kidney transplantation, particularly among highly sensitized candidates. The present study evaluates the system-level impact of this approach, examining transplant probability, allocation dynamics, and the diagnostic performance of epitope-level virtual crossmatching within a large public transplant system.

## Methods

### Study design and setting

We conducted a retrospective cohort study of adult candidates listed for deceased-donor kidney transplantation in Pernambuco, Brazil, comparing two distinct operational periods of the state allocation system. The pre-EpViX era included first registrations from January 2005 to December 2014. The EpViX era comprised first registrations from October 2016 to February 2025. The interval between January 2015 and September 2016 was intentionally excluded, as this period corresponded to pilot testing and progressive implementation of epitope-based allocation strategies.

Eligible participants were adults (≥18 years) listed for deceased-donor kidney transplantation. To avoid duplication of follow-up time, multi-organ listings and repeated registrations within the same era were excluded. Candidates who appeared in both eras were allowed to contribute to each period independently, with era-specific index dates defined by their first registration within that timeframe. The study followed STROBE recommendations for observational research and was approved by the institutional ethics committee.

### Kidney allocation framework in Brazil

Kidney allocation in Brazil is centrally coordinated by the SNT. Candidate ranking is determined by a composite scoring system that incorporates ABO compatibility, HLA matching, waiting time, recipient age, comorbid conditions such as diabetes, prior living donation, and sensitization status. Despite these structured criteria, final eligibility for transplantation remains dependent on a prospective complement-dependent cytotoxicity crossmatch (CDCXM), which serves as the definitive assessment of immunologic compatibility.

### HLA antibody assessment and definition of unacceptable antigens

Anti-HLA antibody assessment was performed using LABScreen assays (One Lambda/Thermo Fisher Scientific). Initial screening was conducted using the LABScreen PRA Class I+II Mixed assay, and reactive samples were subsequently characterized using LABScreen Single Antigen HLA Class I and/or Class II assays. All testing was performed in a single histocompatibility laboratory, ensuring consistency in assay execution and interpretation throughout the study period.

Sera were pre-heated at 56 °C for 1 minute before testing (19). Assay quality-control criteria required negative-control and self-reactivity values below 500 MFI and positive-control values above 5,000 MFI. Antibody monitoring followed Brazilian National Transplant System (SNT) policies. Highly sensitized candidates routinely underwent up to four publicly funded anti-HLA antibody assessments per year, with additional testing performed after sensitizing events when clinically indicated. The EpViX project initially contributed to systematic review and interpretation of antibody profiles, after which monitoring continued according to standard SNT policies.

HLA typing included 11 loci (HLA-A, -B, -C, -DRB1, -DRB3, -DRB4, -DRB5, -DQA1, -DQB1, -DPA1, and -DPB1) and was performed using polymerase chain reaction with sequence-specific oligonucleotide probes (PCR-SSO) at low-to-intermediate resolution; when finer resolution was required to resolve typing ambiguity or to support allele-level unacceptable-antigen assignment, high-resolution typing (Next-Generation Sequencing) was performed. Typing ambiguities were resolved using common and well-documented allele frequencies for the reference population. Consequently, virtual crossmatch assessment incorporated allele-level information derived from the available typing data whenever applicable.

Historical antibody data were actively incorporated into the interpretation process and could contribute to the definition of unacceptable antigens. This approach was adopted because antibody reactivity patterns may fluctuate over time, whereas prior sensitization events may remain clinically relevant even when antibody intensity varies between assessments.

During the EpViX era, antibody interpretation was supplemented by molecular analysis based on eplet definitions from EpRegistry and the conceptual framework proposed by Duquesnoy. Importantly, unacceptable antigens were not assigned solely on the basis of fluorescence intensity. The operational definition of a coherent eplet pattern was the presence of a biologically plausible shared eplet or eplet combination capable of explaining the observed pattern of antibody reactivity across multiple SAB beads.

Accordingly, an antigen was considered unacceptable only when two conditions were simultaneously met: (i) antibody reactivity reached or exceeded an MFI value of 5,000 and (ii) a coherent eplet-based explanation for the observed reactivity pattern could be identified. Reactivities above this threshold without a coherent eplet-based explanation were not automatically assigned as unacceptable antigens. Likewise, reactivities below the predefined threshold were not assigned as unacceptable antigens, irrespective of epitope mapping results.

In instances of allele-specific reactivity, unacceptable antigens were retained at the allele level and were not automatically converted to their corresponding low-resolution antigen equivalents. This strategy ensured that unacceptable antigen assignments were anchored in immunologic plausibility rather than driven solely by signal intensity. Each candidate’s unacceptable antigen profile was subsequently used to calculate cPRA. cPRA was computed from each candidate’s unacceptable-antigen list using the antigen frequencies of the Brazilian deceased-donor reference population, incorporating HLA-A, -B, -C, -DRB1 and -DQB1 loci; the unacceptable-antigen rules (MFI ≥ 5,000 together with a coherent eplet explanation), the incorporation of historical antibodies, the allele-level handling of allele-specific antibodies, and molecular analysis were applied exclusively in the EpViX era. Candidates with cPRA ≥80% were classified as highly sensitized in accordance with Brazilian National Transplant System criteria.

### Epitope-based virtual crossmatch framework

Virtual crossmatching was performed by comparing donor HLA typing with each candidate’s predefined unacceptable antigen profile. A virtual crossmatch was classified as positive when at least one donor antigen matched an unacceptable antigen, and negative otherwise. This framework reflects the conceptual shift introduced by epitope-based models of antibody recognition. The underlying principles were originally described in HLAMatchmaker, which enabled structural interpretation of antibody targets but was not readily scalable for routine clinical use. To address these operational constraints, our group developed EpHLA, an automated platform for epitope reactivity analysis that demonstrated high concordance with HLAMatchmaker while significantly reducing analysis time. Building on this validated foundation, the EpViX was later developed to integrate epitope interpretation with virtual crossmatch execution within a single workflow.

In the present study, EpViX functioned as an analytical support environment. It enabled systematic comparison between donor HLA profiles and recipient unacceptable antigens across the waitlist but did not define the immunologic criteria themselves. Instead, it operationalized a predefined framework based on epitope-informed antibody interpretation.

### Complement-dependent cytotoxicity crossmatch

Prospective complement-dependent cytotoxicity crossmatch (CDCXM) was performed as the final immunologic compatibility assessment before transplantation. Donor lymphocytes were isolated from peripheral blood, spleen, or lymph node samples and separated into T- and B-cell fractions using immunomagnetic cell separation (EasySep™, STEMCELL Technologies). CDCXM was performed separately for T- and B-cell fractions, both with and without anti-human globulin (AHG) augmentation.

For each potential donor–recipient pair, isolated donor lymphocytes were incubated with recipient serum for 30 minutes at room temperature. Crossmatch testing was performed using both untreated and dithiothreitol (DTT)-treated preparations. DTT treatment (30 minutes at 37 °C) was used to reduce interference from IgM antibodies and facilitate identification of clinically relevant IgG-mediated reactivity.

For anti-human globulin–augmented CDCXM (AHG-CDCXM), cell suspensions were washed three times with saline following serum incubation and subsequently incubated with anti-human globulin reagent before addition of rabbit complement. After complement incubation, cell viability was assessed using FluoroQuench staining. Stained cells were evaluated by inverted phase-contrast microscopy and scored according to the proportion of non-viable cells using a standardized scale (0, 2, 4, 6, or 8).

Crossmatch results were considered positive for scores ≥4 and negative for scores ≤2. Reactivity persisting after DTT treatment was interpreted as consistent with IgG antibodies, whereas reactivity observed only in untreated preparations was considered compatible with IgM-mediated reactivity and was not regarded as a contraindication to transplantation. All transplantation decisions remained dependent on prospective CDCXM results in accordance with the allocation workflow.

### Integration into allocation workflow

The unacceptable antigen–based virtual crossmatch was incorporated into the allocation process through the EpViX candidate-selection strategy. EpViX did not replace the existing allocation framework established by the Brazilian National Transplant System (SNT). Rather, it functioned as an additional compatibility-assessment layer operating within the standard allocation process.

Under the Brazilian allocation system, candidates are ranked according to a points-based framework that incorporates ABO compatibility, HLA matching, waiting time, recipient age, diabetes status, prior living kidney donation, sensitization status, and specific statutory priority situations (20). Candidates with cPRA ≥80% receive additional allocation points but do not receive formal priority status within the national allocation policy. Within the Pernambuco program, EpViX-eligible candidates were prioritized for a specific donor offer through the existing statutory prioritization mechanism rather than being permanently moved to the top of the list, and prioritization was applied on a per-donor basis only when the predefined compatibility criteria were met. Displacement of other candidates was constrained by the mandatory negative virtual crossmatch, ABO compatibility, and at least one HLA-DRB1 match, and each prioritization event was recorded and auditable within the allocation system. Final eligibility for transplantation remains dependent on a prospective complement-dependent cytotoxicity crossmatch (CDCXM).

The requirement for at least one HLA-DRB1 match was incorporated into the Pernambuco allocation protocol as an additional immunologic safeguard. This decision was supported by the established weighting of HLA-DR matching within the Brazilian allocation system, the available literature at the time of implementation, and allocation strategies adopted in Eurotransplant programs. The criterion was intended to balance expanded transplant access for highly sensitized candidates with maintenance of a minimum level of immunologic compatibility and to reduce the risk that epitope-based candidate selection combined with ABO-compatible allocation could result in excessive displacement of candidates within the standard ranking system.

Candidates identified as eligible by EpViX remained subject to all standard allocation requirements, including ABO compatibility, ranking criteria established by the SNT, and prospective physical crossmatch testing before transplantation.

Subsequent clinical evaluation and prospective CDCXM were performed for all potential transplant recipients. Transplantation proceeded only when virtual and physical crossmatch results were concordantly negative. Accordingly, virtual crossmatching functioned as a screening and compatibility-assessment tool rather than a replacement for laboratory confirmation.

### Statistical analysis

Continuous variables were summarized as medians with interquartile ranges (IQR) and compared using the Mann–Whitney U test. Categorical variables were expressed as counts and percentages and compared using the χ² or Fisher’s exact tests, as appropriate.

Time to transplantation was evaluated using Kaplan–Meier methods, with differences between eras assessed using the log-rank test. Multivariable Cox proportional hazards models were used to estimate hazard ratios (HRs) and 95% confidence intervals (CIs). Robust standard errors clustered at the patient level were applied to account for candidates who contributed observations in both study eras.

The proportional hazards assumption was evaluated using Schoenfeld residuals. Because evidence of non-proportional hazards was identified, additional analyses were performed to characterize time-varying effects. These included estimation of restricted mean survival time (RMST) differences and interval-specific hazard ratios. Absolute adjusted probabilities of transplantation at prespecified time points were also calculated to facilitate clinical interpretation of the observed associations.

To evaluate the robustness of the findings, several sensitivity analyses were performed. First, analyses were repeated using a restricted time window comparing candidates listed between 2011–2014 and 2016–2019. Second, within-era deduplication analyses were conducted by restricting the dataset to a single registration per candidate within each era. Third, interrupted time-series analyses were performed using segmented negative-binomial regression of the monthly transplantation rate (with an offset for person-time at risk; the negative-binomial family was used to accommodate overdispersion in the monthly event counts), excluding the implementation period, to assess temporal changes associated with introduction of the EpViX allocation strategy.

The number of prioritization events per candidate was analyzed using Poisson regression with robust variance estimation, and results are reported as incidence rate ratios (IRRs) with 95% confidence intervals.

Diagnostic performance of the epitope-level virtual crossmatch (eVXM) was assessed against prospective complement-dependent cytotoxicity crossmatch (CDCXM), using individual crossmatch tests as the unit of analysis. Results were summarized using a 2×2 contingency framework, allowing classification as true positive, true negative, false positive, or false negative. Accuracy, sensitivity, specificity, positive predictive value (PPV), and negative predictive value (NPV) were calculated with 95% confidence intervals estimated using the Wilson method. Because eVXM was intended to function as a screening tool within the allocation workflow, particular emphasis was placed on sensitivity and NPV. Because multiple crossmatch assays were performed per donor and per candidate, the assays were not statistically independent; therefore, the reported 95% confidence intervals are descriptive, assay-level estimates that do not account for clustering by candidate or donor and should be interpreted as such.

The administrative dataset contained information on transplantation status and date of last follow-up but did not provide complete and consistently validated information regarding specific reasons for waitlist exit across both eras. Consequently, candidates who did not undergo deceased-donor transplantation were censored at their last recorded follow-up date. Because competing events such as death, living-donor transplantation, delisting, transfer, or inactive status could not be reliably identified throughout the study period, competing-risk analyses were not performed. This limitation is acknowledged in the interpretation of the time-to-event analyses.

### Missing data

Missing data were quantified by era and for each analysis variable (**Supplementary Table 1**). Candidate-level missingness was 0% in the pre-EpViX era and ≤ 0.13% in the EpViX era; donor-level and allocation variables were available only for the EpViX era, and no imputation was performed.

Several donor-level variables, including donor age, donor sex, donor ABO blood group, and DRB1 mismatch, were unavailable for the pre-EpViX era because these fields were not collected in the historical registry system. These variables were therefore absent by data provenance rather than missing at random and were not incorporated into the primary comparative analyses.

## Results

### System-level impact of EpViX and prioritization

**Figure 1:**
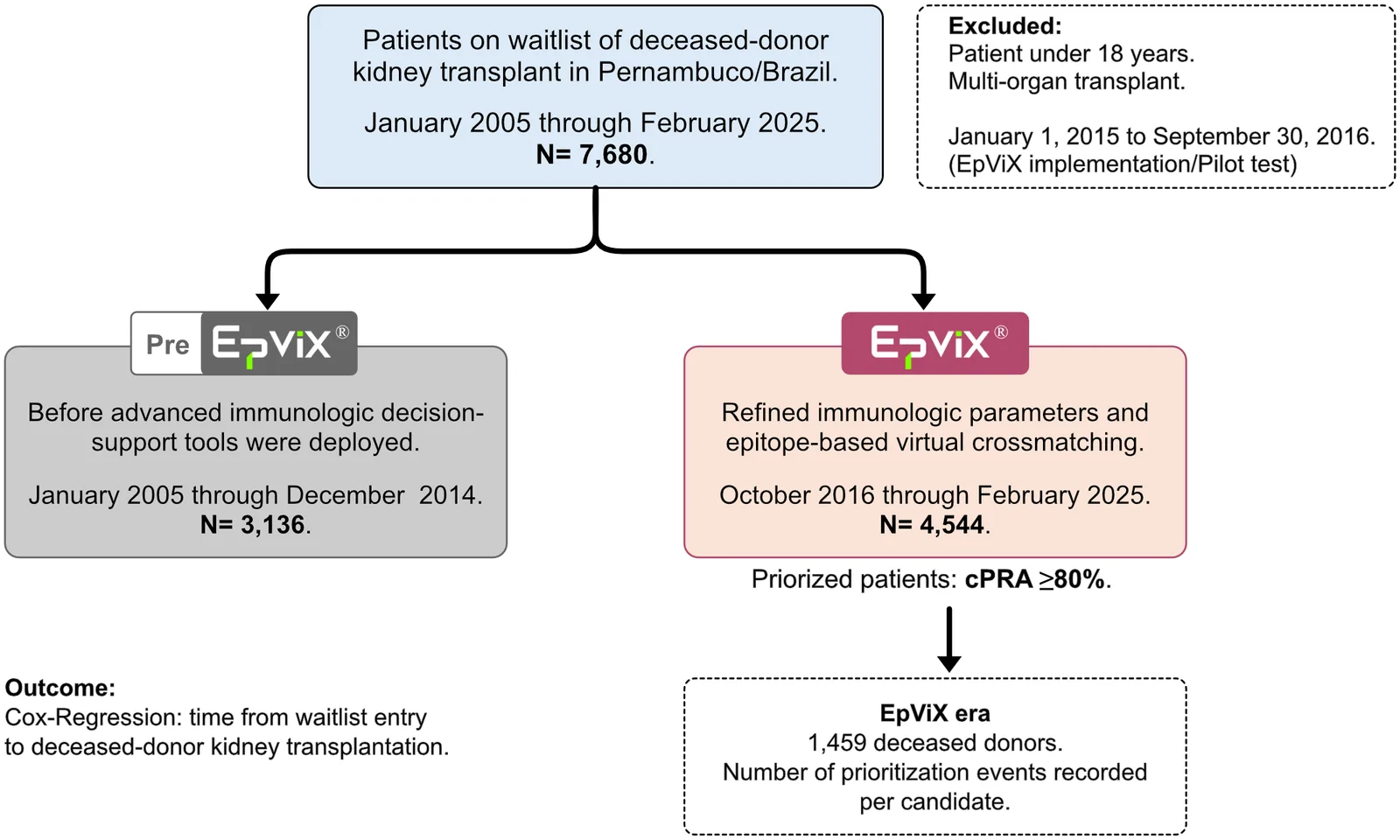
Study flowchart. Cohort assembly across the pre-EpViX and EpViX eras.

We analyzed 7,680 adult candidates listed for deceased-donor kidney transplantation in Pernambuco across two distinct eras: 3,136 candidates in the pre-EpViX era and 4,544 candidates in the EpViX era. The cohort assembly process is detailed in **Figure 1**.

### Baseline characteristics and outcomes

Baseline characteristics and outcomes varied between the pre-EpViX (2005–2014) and EpViX (2016–2025) eras (**Table 1**). Although the sex distribution remained stable, with males accounting for approximately 60% of listed candidates in both periods (*p* = 0.6), other parameters - including donor origin, sensitization profile, and transplant outcomes - showed significant shifts.

**Table 1:** Baseline characteristics and outcomes of deceased-donor kidney transplant candidates in Pernambuco, comparing the pre-EpViX era (2005-2014) with the EpViX era (2016-2025).

Characteristic	pre-EpViX, N = 3,136	EpViX, N = 4,544	p-value
Sex, n (%)			
Female	1,253 (40%)	1,790 (39.39%)	0.6
Male	1,883 (60%)	2,752 (60.56%)	
Unknow	0 (0%)	2 (0.05%)	
Donor area, n (%)			
Other states	747 (24%)	910 (20%)	<0.001
Pernambuco state	2,389 (76%)	3,634 (80%)	
**cPRA, median (IQR)**	0 (0, 13)	0 (0, 0)	<0.001
cPRA category, n (%)			
0%	2,255 (72%)	3,479 (76.56%)	<0.001
1-79%	553 (18%)	700 (15.41%)	
80-94%	140 (4.5%)	185 (4.07%)	
95-100%	188 (6.0%)	174 (3.83%)	
Unknow	0 (0%)	6 (0.13%)	
**Highly sensitized (cPRA ≥80%), n (%)**	328 (10%)	359 (7.9%)	<0.001
**Time to transplant among transplanted candidates, months, median (IQR) <0.001**	22 (10, 43)	7 (2, 17)	<0.001
**Transplanted during follow-up, n (%)**	1,126 (36%)	2,253 (50%)	<0.001
**Lack-of-access priority, n (%)**	59 (1.9%)	218 (4.8%)	<0.001

cPRA, calculated panel-reactive antibody; IQR, interquartile range; “Donor area” indicates the geographic origin of the kidney offer; “Lack-of-access priority” indicates assignment to the statutory priority class for candidates with prolonged waiting times. Waiting time to transplantation was calculated only among candidates who underwent deceased-donor kidney transplantation.

The geographic origin of donor kidneys demonstrated a progressive trend toward local procurement. The proportion of organs originating from within Pernambuco increased from 76% in the pre-EpViX era to 80% during the EpViX period (*p* < 0.001), reflecting the growing regionalization of the donor pool and potentially improved coordination among procurement organizations.

Sensitization profiles also evolved markedly between eras. Median cPRA values remained at 0 across both periods; however, the interquartile range narrowed substantially - from 0–13 in the pre-EpViX era to 0–0 during the EpViX era (*p* < 0.001) - indicating a population-wide shift toward lower overall sensitization and reduced variability among candidates. The proportion of candidates with cPRA =0% increased from 72% to 77% (*p* < 0.001), while the proportion of highly sensitized candidates (cPRA ≥80%) declined from 10.0% to 7.9% (*p* < 0.001), with reductions across both sensitization strata: from 4.5% to 4.1% in the 80-94% range and from 6.0% to 3.8% in the 95-100% range.

Access to transplantation was higher during the EpViX era. The proportion of candidates who received a deceased-donor kidney increased from 36% to 50% (*p* < 0.001), representing a 39% relative increase in realized transplants. Among transplanted patients, median waiting time fell sharply from 22 months (IQR 10-43) in the pre-EpViX era to 7 months (IQR 2-17) during the EpViX era (*p* < 0.001).

Furthermore, the proportion of candidates assigned to the statutory “lack-of-access” priority category more than doubled - from 1.9% in the pre-EpViX period to 4.8% in the EpViX era (*p* < 0.001). This finding indicates a greater frequency of assignment to the statutory lack-of-access priority category during the EpViX era.

### Overall impact on access to transplantation

**Figure 2:**
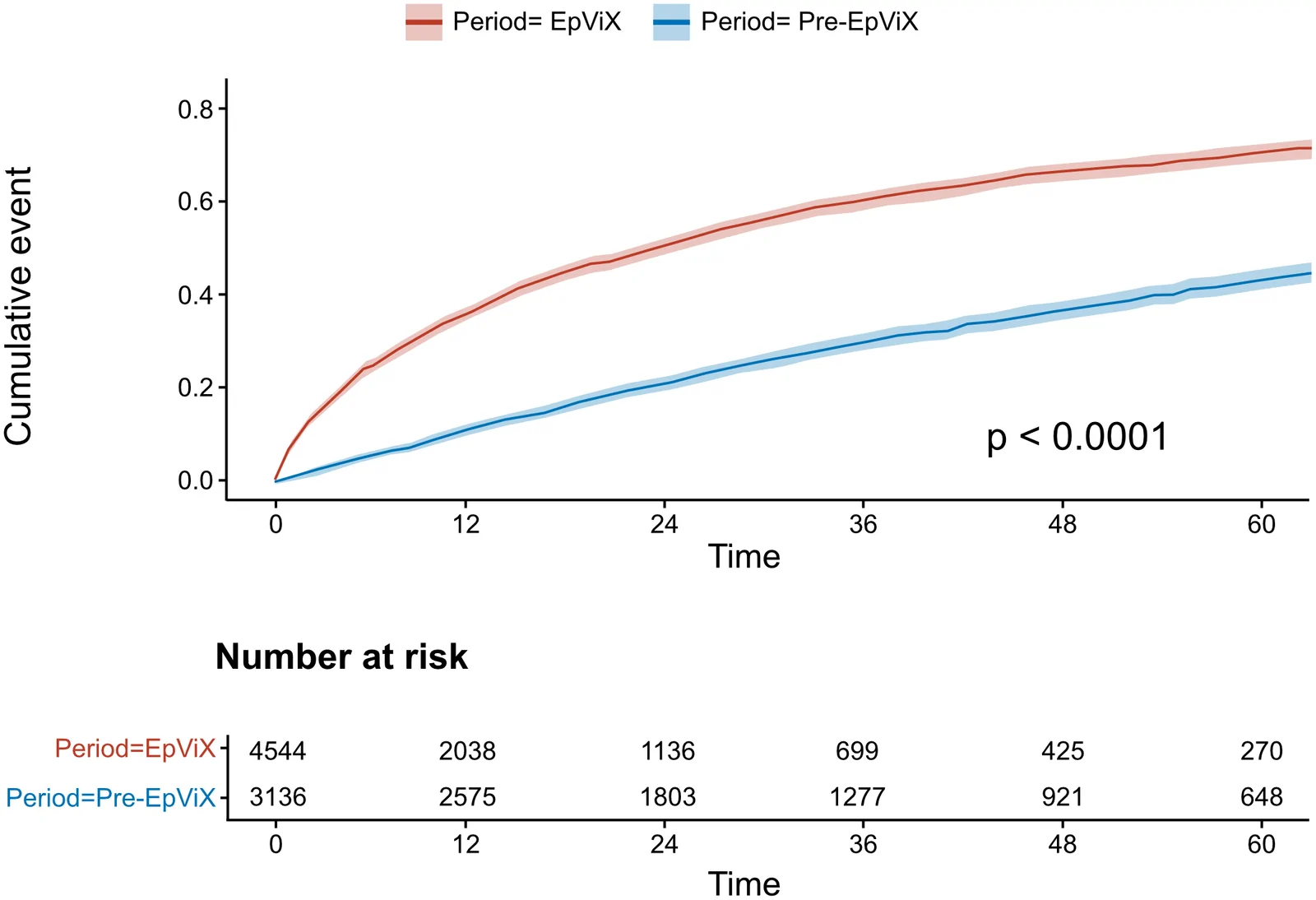
Time to transplantation by era. Kaplan–Meier curves for the full waitlisted population comparing pre-EpViX vs EpViX.

Kaplan–Meier analyses revealed a markedly higher cumulative incidence of transplantation in the EpViX era compared with the pre-EpViX period (**Figure 2**). At 12 months, the cumulative incidence reached 11% (95% CI, 10-12%) in the pre-EpViX era versus 36% (95% CI, 35-38%) during the EpViX era (*p* < 0.001, log-rank test). By 60 months, these rates increased to 43% (95% CI, 41-45%) and 70% (95% CI, 68-72%), respectively, highlighting a sustained improvement in transplant access over time.

In adjusted Cox proportional-hazards models, listing in the EpViX era was independently associated with a 2.55-fold higher hazard of transplantation compared with the pre-EpViX era (HR = 2.55; 95% CI, 2.37-2.74; *p* < 0.001) after adjustment for sensitization (cPRA) and lack-of-access priority (**Table 2**). Sensitization remained a strong inverse predictor of transplantation probability. Relative to unsensitized candidates (cPRA = 0%), hazard ratios decreased progressively with higher sensitization: 0.62 (95% CI, 0.55-0.68; *p* < 0.001) for cPRA 1–79%, 0.54 (95% CI, 0.44-0.66; *p* < 0.001) for cPRA 80-94%, and 0.18 (95% CI, 0.13-0.24; *p* < 0.001) for cPRA 95-100%. The proportional-hazards assumption was not met for the era effect (scaled Schoenfeld residuals, global *p* < 0.001); the EpViX association was strongest in the first year (HR 3.97, 95% CI 3.52-4.46 over 0–12 months) and attenuated thereafter (HR 1.74, 95% CI 1.57-1.93), and over a 60-month horizon candidates reached transplantation on average about 16 months sooner in the EpViX era. Covariate-standardized absolute probabilities of transplantation were 15%, 31% and 41% (pre-EpViX) versus 34%, 60% and 73% (EpViX) at 12, 36 and 60 months (**Supplementary Table 2**). The association was preserved when restricting to listings nearer implementation (HR 2.17, 95% CI 1.98-2.39) and when excluding within-era duplicate registrations (HR 2.31, 95% CI 2.14-2.49). In the interrupted time-series, EpViX implementation was associated with an immediate increase in the transplantation rate (level-change IRR 1.89, 95% CI 1.06-3.36; *p* = 0.03) over and above a pre-existing upward secular trend, although residual autocorrelation and the differing observation windows of the two data sources warrant cautious interpretation.

**Table 2:** Multivariable Cox proportional hazards model for time to transplantation among all waitlisted candidates (n=7,680).

Multivariable Cox model	Hazard ratio	95% CI	p-value
Model 1 (Main effects)			
Pre-EpViX	1.00 (ref)	—	—
EpViX	2.55	2.37–2.74	<0.001
cPRA category			
0%	1.00 (ref)	—	—
1-79%	0.62	0.55-0.68	<0.001
80-94%	0.54	0.44-0.66	<0.001
95-100%	0.18	0.13-0.24	<0.001
Lack-of-access priority			
No	1.00 (ref)	—	—
Yes	1.48	1.24-1.77	<0.001
Model 2 (Interaction): Model 1 plus era × highly sensitized			
Pre-EpViX unsensitized	1.00 (ref)	—	—
EpViX unsensitized	2.72	2.53-2.94	<0.001
Pre-EpViX HS (cPRA ≥80)	0.47	0.37-0.60	<0.001
EpViX HS (cPRA ≥80)	0.54	0.36-0.82	0.004

Model adjusted for sensitization (cPRA) and lack-of-access priority status; donor region was excluded as a post-baseline variable, and transplant center was not available in the present dataset. Robust standard errors clustered at patient level. cPRA, calculated panel-reactive antibody.

The interaction model (Model 2) demonstrated that candidates listed during the EpViX era exhibited higher transplantation rates across all sensitization strata. Although highly sensitized (HS) candidates remained disadvantaged compared with unsensitized recipients from the pre-EpViX era, those listed during the EpViX period exhibited a 15% higher transplantation hazard than their pre-EpViX HS counterparts (HR = 0.54 vs 0.47). These findings indicate a partial narrowing of the sensitization gap under the EpViX allocation system.

**Figure 3:**
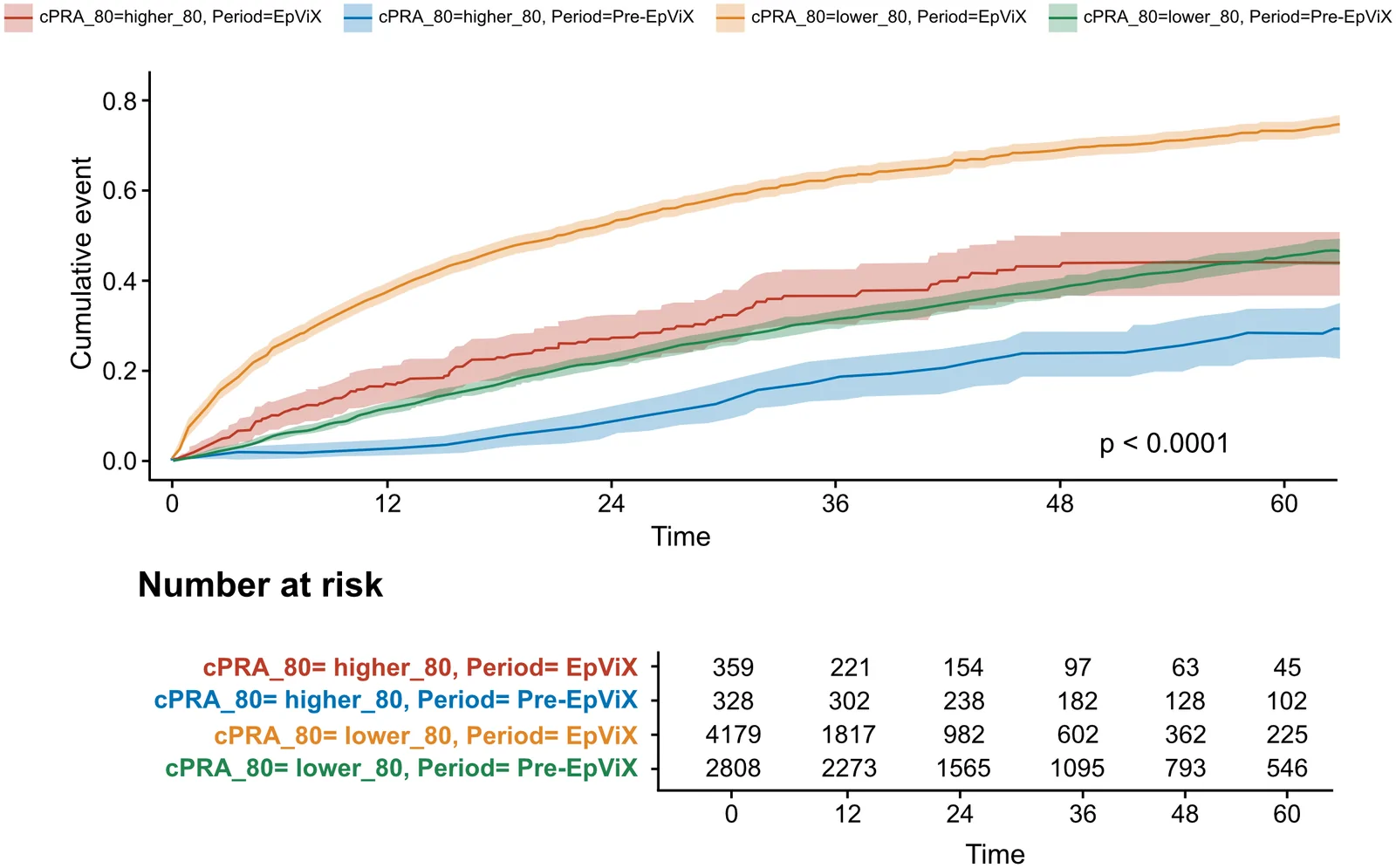
Time to transplantation by sensitization within each era. Era-specific Kaplan–Meier curves stratified by cPRA <80% vs cPRA ≥80% (pre-EpViX and EpViX).

Kaplan–Meier curves stratified by both era and sensitization status (cPRA <80% vs cPRA ≥80%) further illustrate these improvements (**Figure 3**). Among non-sensitized candidates, the EpViX era was associated with substantially enhanced access to transplantation, with cumulative incidence increasing from 12% to 38% at 12 months and from 45% to 73% at 60 months (*p* < 0.001, log-rank test). Similarly, among highly sensitized candidates, cumulative incidence rose from 3% to 17% at 12 months and from 28% to 44% at 60 months (*p* < 0.001). Together, these results are consistent with greater access to transplantation during the EpViX era to deceased-donor kidney transplantation across the entire candidate population, including those historically disadvantaged by high immunologic sensitization.

### Donor-episode analysis

During the EpViX era, 1,459 deceased donors generated kidney offers that were analyzed by the system. Prioritization occurred in 130 of 1,459 donor episodes (8.9%), including 67 of 83 episodes involving highly sensitized recipients (81%). Donor-level outcomes and recipient characteristics stratified by recipient sensitization status are shown in **Table 3**. Both kidneys were transplanted from 818 donors (56%), one kidney from 612 donors (42%), and no kidneys from 29 donors (2.0%). When highly sensitized recipients received transplants (n=83 donor episodes, 5.7% of total), both kidneys were more frequently utilized compared with episodes involving non-sensitized recipients only: 72% versus 55% for two-kidney utilization (*p* < 0.001).

**Table 3:** Donor-episode characteristics in the EpViX era, stratified by recipient sensitization status (n=1,459 donors).

Characteristic	Overall, N = 1,459	HS recipients, N = 83	NHS recipients, N = 1,376	p-value
Donor region, n (%)				<0.001
Other states	624 (43%)	13 (16%)	611 (44%)	
Pernambuco state	835 (57%)	70 (84%)	765 (56%)	
Kidneys transplanted per donor, n (%)				<0.001
0	29 (2.0%)	4 (4.8%)	25 (1.8%)	
1	612 (42%)	19 (22.9%)	593 (43.1%)	
2	818 (56%)	60 (72.3%)	758 (55.1%)	
**Time from offer to allocation, months, median (IQR)**	9 (3, 17)	13 (6, 23)	8 (3, 17)	0.003
**Eplet mismatches DRB1, median (IQR)**	8 (3, 15)	10 (3, 15)	8 (3, 15)	0.4
**Eplet mismatches DQ, median (IQR)**	9.0 (4.0, 13.0)	9.0 (3.3, 12.5)	9.0 (4.0, 13.0)	0.6
**Absolute waitlist position, median (IQR)**	5 (3, 17)	72 (10, 285)	5 (3, 12)	<0.001
**Recipient cPRA, median (IQR)**	0 (0, 8)	46 (34, 88)	0 (0, 1)	<0.001
Recipient cPRA category, n (%)				<0.001
0%	1,215 (83.3%)	0 (0%)	1,215 (88%)	
1-79%	161 (11%)	0 (0%)	161 (12%)	
80-94%	66 (4.5%)	66 (80%)	0 (0%)	
95-100%	17 (1.2%)	17 (20%)	0 (0%)	
**At least one kidney transplanted, n (%)**	1,430 (98%)	79 (95%)	1,351 (98%)	0.078
**Prioritization applied, n (%)**	130 (8.9%)	67 (81%)	63 (4.6%)	<0.001

cPRA, calculated panel-reactive antibody; DQ, HLA-DQ locus; DRB1, HLA-DRB1 locus; HS, highly sensitized (cPRA ≥80%); NHS, non-highly sensitized (cPRA <80%); IQR, interquartile range. Analysis performed at donor-episode level, each donor episode may result in 0, 1, or 2 transplants; “HS Recipients” indicates donor episodes in which at least one recipient had cPRA ≥80%.

Highly sensitized recipients had higher median cPRA (46% vs 0%, *p* < 0.001) and substantially worse absolute waitlist positions (median rank 72 vs 5, *p* < 0.001), consistent with prioritization of candidates who otherwise occupied substantially lower positions on the waitlist. Contrary to potential concerns that prioritizing highly sensitized candidates might require acceptance of higher immunologic mismatching, eplet mismatch loads were comparable between groups. Median eplet mismatches at HLA-DRB1 were 10 (IQR 3-15) for highly sensitized recipients versus 8 (IQR 3-15) for non-sensitized recipients (*p* = 0.4). Similarly, at HLA-DQ (DQ), median mismatches were 9.0 (IQR 3.3-12.5) versus 9.0 (IQR 4.0-13.0), respectively (*p* = 0.6).

Highly sensitized recipients experienced modestly longer times from donor offer to final allocation, with a median of 13 months (IQR 6-23) versus 8 months (IQR 3-17) for non-sensitized recipients (*p* = 0.003).

### Prioritization intensity by sensitization level

To quantify how frequently the EpViX prioritization strategy selected candidates across different sensitization strata, we modeled the count of prioritization events per candidate using Poisson regression (**Table 4**). Using unsensitized candidates (cPRA =0%) as the reference, candidates with cPRA 1-79% had fewer prioritization events (IRR 0.21, 95% CI 0.13-0.33, *p* < 0.001), consistent with the algorithm focus on highly sensitized candidates. In contrast, highly sensitized candidates experienced markedly higher prioritization rates. Candidates with cPRA 80-94% had 8.65 times more prioritization events than unsensitized candidates (IRR 8.65, 95% CI 4.58-16.35, *p* < 0.001). Candidates with cPRA 95-100% had 3.38 times more prioritization events (IRR 3.38, 95% CI 1.62-7.07, *p* < 0.001).

**Table 4:** Prioritization intensity by sensitization level: Poisson regression number of EpViX prioritization events per candidate.

cPRA category	IRR	95% CI	p-value
0%	1.00(ref)	—	
1-79%	0.21	0.13, 0.33	<0.001
80-94%	8.65	4.58, 16.35	<0.001
95-100%	3.38	1.62, 7.07	<0.001

Model adjusted for sensitization (cPRA). Robust (sandwich) variance estimation used to account for clustering and overdispersion; reported confidence intervals reflect the robust estimator. IRR >1 indicates more prioritization events relative to reference group; IRR <1 indicates fewer events; cPRA, calculated panel-reactive antibody.

### Diagnostic performance of epitope virtual crossmatch

**Figure 4:**
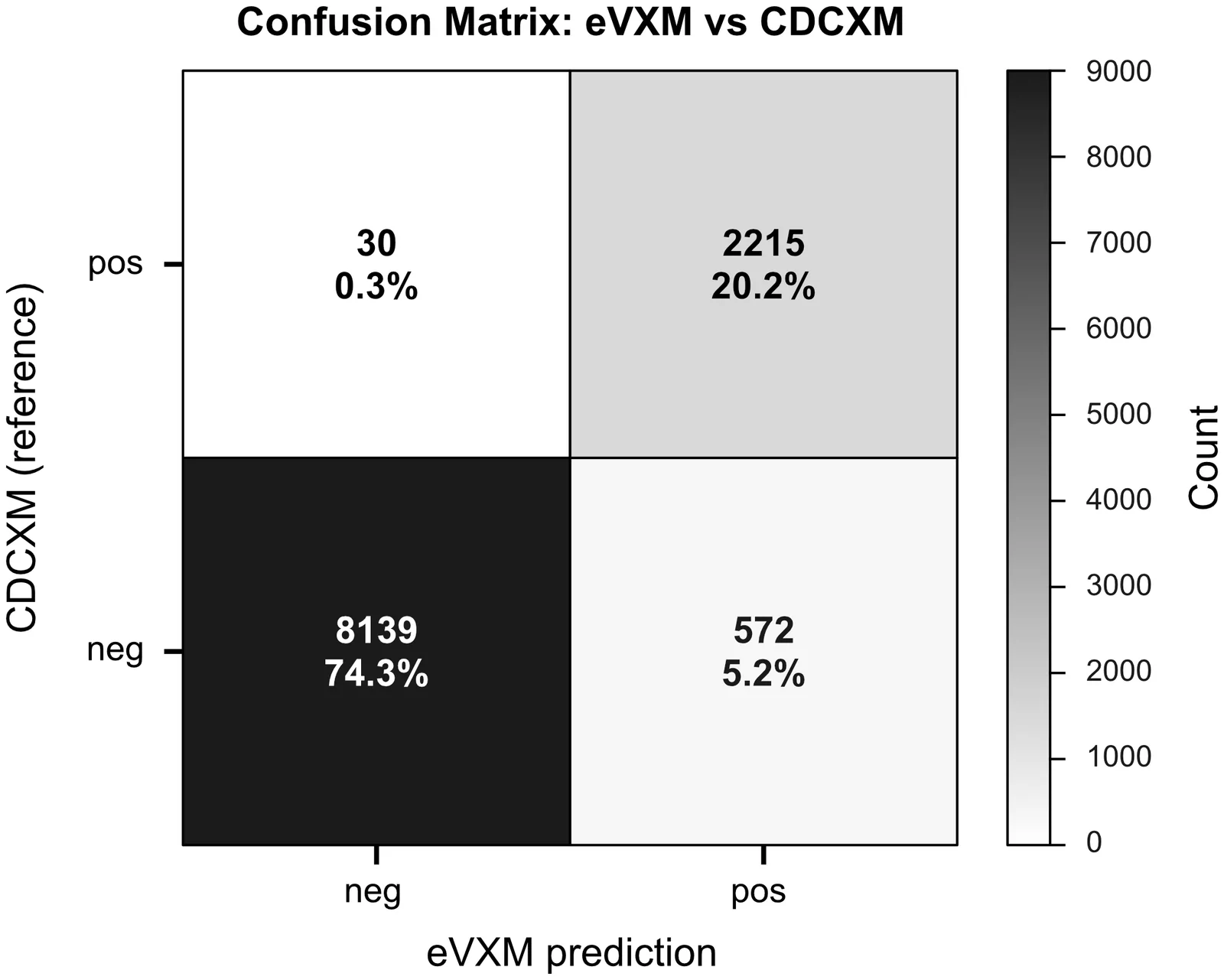
Confusion matrix comparing epitope-level virtual crossmatch (eVXM) and complement-dependent cytotoxicity crossmatch (CDCXM). The matrix summarizes the results of 10,956 crossmatch assays performed during the EpViX era, reflecting the allocation workflow in which multiple crossmatches are generated per donor against different potential recipients. Concordant negative results (true negatives) and concordant positive results (true positives) represent agreement between eVXM and CDCXM, whereas discordant cells indicate false-positive eVXM results (eVXM positive, CDCXM negative) and false-negative eVXM results (eVXM negative, CDCXM positive). Values are shown as absolute counts and percentages of all crossmatch assays.

We evaluated the agreement between the eVXM performed by EpViX and the prospective CDCXM using 10,956 crossmatch assays performed during the EpViX era (**Figure 4**). These assays reflect the operational allocation workflow, in which multiple crossmatches are performed per donor, typically against a panel of potential recipients.

The confusion matrix demonstrated a predominance of CDCXM-negative assays. Of all crossmatches analyzed, 8,139 (74.3%) were concordantly negative by both eVXM and CDCXM, representing true negatives, whereas 2,215 assays (20.2%) were concordantly positive and classified as true positives. Discordant results included 572 false-positive eVXM assays (5.2%), in which eVXM predicted incompatibility despite a negative CDCXM result, and 30 false-negative assays (0.3%), in which eVXM failed to predict CDCXM positivity. A qualitative review of these 30 false-negative assays, grouped by the most likely explanation, is summarized in **Supplementary Table 3**.

Overall diagnostic accuracy across all crossmatch assays was 94.5% (95% CI, 94.1–94.9). The eVXM demonstrated a sensitivity of 98.7% (95% CI, 98.1–99.1), correctly identifying nearly all positive CDCXM results, and a specificity of 93.4% (95% CI, 92.9–93.9), indicating robust identification of negative CDCXM results.

The NPV was 99.6% (95% CI, 99.5–99.7), underscoring the reliability of a negative eVXM result for ruling out CDCXM positivity at the crossmatch level. In contrast, the PPV was 79.5% (95% CI, 77.9–80.9), reflecting the conservative nature of epitope-based prediction and the expected presence of false-positive results in a screening context.

## Discussion

The implementation of an allocation strategy grounded in the definition of unacceptable antigens and informed by epitope-level antibody interpretation represents a meaningful shift in how immunologic compatibility is approached within a public transplant system. In the present cohort, this approach was associated with greater access to deceased-donor kidney transplantation. These findings were observed across the allocation system, with particularly relevant gains among highly sensitized candidates and high concordance between virtual and prospective physical crossmatch testing.

Before the introduction of this framework, the experience in Pernambuco closely mirrored that described in other regions. Highly sensitized candidates consistently faced longer waiting times and lower probabilities of transplantation, even in allocation systems that incorporated prioritization policies (21–24). Similar disparities have also been documented in Brazilian cohorts, where sensitization remains a strong determinant of reduced access despite adjustments in allocation scoring (25, 26). Taken together, these observations highlight a persistent limitation of strategies that rely primarily on antigen-level matching, without incorporating a more detailed understanding of antibody reactivity.

The present findings suggest that redefining compatibility through unacceptable antigens, informed by epitope-level interpretation, can partially overcome these structural barriers. When antibody reactivity is interpreted at the level of epitopes, it becomes possible to distinguish between patterns that are likely to be clinically relevant and those that are not. In practical terms, this approach may expand the pool of potential donors while maintaining consistency with predefined immunologic compatibility criteria. This interpretation is consistent with prior work showing that epitope-based approaches more accurately reflect alloimmune risk than conventional antigen-level matching (27–29).

The magnitude of the system-level changes observed here is notable. The proportion of candidates undergoing transplantation increased substantially, and median waiting time decreased in parallel. These improvements were not confined to highly sensitized candidates, suggesting that the incorporation of epitope-based compatibility did not simply redistribute access within the waitlist. Instead, it was associated with higher transplantation rates across the waitlist. This interpretation is supported by the Kaplan–Meier analyses and by the adjusted Cox models, in which listing in the EpViX era remained strongly associated with a higher hazard of transplantation.

At the same time, sensitization continued to exert a negative effect on transplant probability. This finding is expected and aligns with a broad body of literature demonstrating the impact of anti-HLA antibodies on access to transplantation (30, 31). What is notable, however, is the relative improvement observed among highly sensitized candidates in the EpViX era. Although these individuals remain disadvantaged compared with non-sensitized candidates, the gap appears to have narrowed. Similar patterns have been reported following allocation reforms in other settings, including the Kidney Allocation System in the United States (23, 32).

The donor-episode analysis provides an additional perspective on how these changes manifest in practice. The higher frequency of two-kidney utilization in donor episodes involving highly sensitized recipients suggests that epitope-informed compatibility assessment can reveal opportunities that might otherwise remain undetected. This observation is consistent with reports from acceptable mismatch and epitope-based allocation programs, where broader definitions of compatibility have been associated with improved organ utilization (21, 28, 31). Importantly, this expansion did not appear to come at the cost of increased immunologic mismatch, as eplet mismatch loads were comparable between sensitized and non-sensitized recipients. This finding aligns with previous studies indicating that epitope-based allocation can maintain immunologic compatibility while expanding access to transplantation (27, 33).

The diagnostic performance of the eVXM further reinforces its role within this framework. The very high NPV observed in this study indicates that a negative virtual crossmatch result reliably excludes CDCXM-positive outcomes. From a clinical perspective, this is precisely the characteristic required for a screening tool in the allocation process. These findings are in line with prior reports demonstrating the diagnostic reliability and utility of virtual crossmatching in renal transplantation (34–36). The presence of false-positive results, while expected, reflects a conservative approach and supports the continued use of confirmatory physical crossmatch testing before transplantation.

Another important aspect of the allocation strategy is its proportionality with respect to sensitization level. The prioritization analysis shows that candidates with intermediate levels of sensitization experienced the greatest increase in prioritization intensity, while those with the highest levels of sensitization also benefited, albeit to a lesser extent. This pattern is consistent with prior observations suggesting that moderate sensitization may offer more opportunities for compatible matching than extreme sensitization, likely due to underlying haplotype frequency constraints (22, 30, 37).

Beyond the immunologic domain, the increase in locally sourced donors during the EpViX era suggests improvements in system organization and coordination. Efficient donor identification, comprehensive HLA typing, and timely integration of immunologic data are all recognized prerequisites for the successful implementation of epitope-based allocation strategies (28, 38). Implementation at scale requires substantial established infrastructure, including comprehensive donor and recipient HLA typing, single-antigen-bead antibody testing, dedicated immunogenetics expertise for eplet-level interpretation, software support (EpViX or similar), and integration with allocation logistics; the scalability of this approach should be interpreted in light of these requirements. The experience in Pernambuco illustrates that these elements can be effectively combined within a publicly funded healthcare system (39). These observations become particularly relevant when considering more sensitive physical crossmatch methodologies.

The eVXM performance should also be interpreted in the context of more sensitive physical crossmatch methodologies. Although CDCXM was the reference assay used in the present allocation workflow, flow-cytometry crossmatch (FXM) is known to detect lower levels of donor-specific antibody reactivity and may further refine immunologic risk assessment. In this regard, the relatively modest positive predictive value observed for eVXM should not necessarily be interpreted as evidence of clinically unacceptable risk. Previous studies have shown that a proportion of candidates with donor-specific antibodies identified by solid-phase assays remain FXM-negative and may experience acceptable post-transplant outcomes (40–43). These observations highlight the importance of defining operational thresholds for unacceptable antigens carefully, balancing immunologic risk against unnecessary restrictions in donor access. Excessively conservative thresholds may reduce transplantation opportunities without necessarily identifying risk levels that translate into adverse clinical outcomes.

The increasing adoption of virtual crossmatch strategies worldwide reflects this evolving perspective (34, 41, 44–48). In contemporary allocation systems, virtual crossmatching is increasingly used as an efficient screening and candidate-selection tool, while prospective CDCXM or FXM remain important confirmatory assessments before transplantation. From an implementation perspective, the major challenges are often not technological but operational, including establishment of consensus policies for antibody acceptability, management of limitations inherent to solid-phase assays, and training in molecular interpretation of antibody reactivity patterns. The Pernambuco experience suggests that these challenges can be successfully addressed within a publicly funded transplant program. Several limitations should be considered when interpreting these findings. The study was conducted in a single state and reflects the specific infrastructure and practices of that setting. Although multivariable adjustment was performed, residual confounding cannot be excluded, particularly in relation to center-level practices and temporal changes in donor characteristics or clinical management (23, 49). A center-stratified sensitivity analysis was planned; however, a validated transplant-center identifier was not available in the administrative dataset, so this analysis could not be performed, and center-level practice changes cannot be fully excluded as contributors to the observed era effect. In addition, competing risks such as death or delisting were not explicitly modeled, which may influence estimates of time to transplantation. Finally, long-term post-transplant outcomes were not evaluated. The relationship between epitope mismatch and chronic alloimmune injury remains complex and continues to be an area of active investigation (33, 50). Several features temper causal interpretation. Because the eras are defined by non-overlapping calendar periods, calendar time could not be fully separated from the intervention; reassuringly, the association persisted in analyses restricted to years nearer implementation and in interrupted time-series. Competing events such as death on the list, delisting and living-donor transplantation could not be classified consistently across eras, so competing-risk models were not estimable; in the presence of competing events, Kaplan–Meier methods may overestimate the absolute probability of deceased-donor transplantation, and the direction of bias in the between-era comparison is uncertain. Finally, the two eras originate from different registry systems with non-interoperable identifiers, so individuals contributing to both periods could not be enumerated or linked, although a sensitivity analysis excluding within-era duplicates left estimates essentially unchanged.

In summary, these finds support the integration of unacceptable antigen–based, epitope-informed compatibility assessment into kidney allocation systems. The EpViX experience in Pernambuco demonstrates that implementation of this strategy is feasible within a publicly funded transplant program and was associated with greater access to deceased-donor kidney transplantation, including among highly sensitized candidates. Further studies incorporating post-transplant outcomes and external validation in other settings will be important to determine the broader clinical impact of this approach.

## Conclusions

The EpViX experience in Pernambuco suggests that integration of epitope-informed compatibility assessment into kidney allocation workflows is feasible within a publicly funded transplant system and is associated with greater access to deceased-donor kidney transplantation. Further studies evaluating long-term post-transplant outcomes and implementation in other settings will be important to determine the broader clinical impact of this approach.

## Data Availability

The raw data supporting the conclusions of this article will be made available by the authors, without undue reservation.
