# Supplementary material for: Retrospective cohort study of epitope-based virtual crossmatch allocation in a public transplant system (Pernambuco, Brazil)
Source: Front Immunol. 2026 Jul 28;17:1879817. doi: 10.3389/fimmu.2026.1879817 (PMC13491654; doi:10.3389/fimmu.2026.1879817)
Supplement: Supplementary file 1 [file DataSheet1.pdf]

SUPPLEMENTARY MATERIAL

Supplementary Table S1. Missing data by era and analysis variable.

Variable	Pre-EpViX (n = 3,136)	EpViX (n = 4,544)
Candidate-level variables		
Era / waitlist-entry date	0 (0%)	0 (0%)
Time to event (used in survival models)	0 (0%)	0 (0%)
Transplant outcome	0 (0%)	0 (0%)
Sensitization (cPRA)	0 (0%)	6 (0.13%)
Lack-of-access priority status	0 (0%)	0 (0%)
Recipient sex	0 (0%)	2 (0.04%)
Recipient ABO blood group	0 (0%)	5 (0.11%)
Donor-level and allocation variables		
Donor age	—[a]	24 (0.53%)
Donor sex	—[a]	13 (0.29%)
Donor ABO blood group	—[a]	5 (0.11%)
DRB1 (epitope) mismatch	—[a]	9 (0.20%)
Allocation rank position	—[a]	0 (0%)

Values are number of candidates with missing data (percentage of the era denominator). cPRA, calculated panel-reactive antibody; ABO, ABO blood group; DRB1, HLA-DRB1 locus.

[a] Not provided by the national transplant registry (Sistema Nacional de Transplantes) for the pre-EpViX era, in which only candidate-registration and transplantation/exit data were available; these fields are absent by data provenance rather than missing at random.

No imputation was performed. All models used complete cases; given the negligible candidate-level missingness (≤ 0.13% in either era), this had no material effect on the estimates.

Supplementary Table S2. Proportional-hazards diagnostics, time-varying effects, adjusted absolute probabilities, and sensitivity analyses for the association between allocation era and time to deceased-donor transplantation.

Analysis	Estimate (95% CI)
**Panel A. Proportional-hazards test (scaled Schoenfeld residuals)**	
EpViX era	$\chi^2 = 145.1$, df = 1, $p < 0.001$
Sensitization (cPRA category)	$\chi^2 = 14.0$, df = 3, $p = 0.003$
Lack-of-access priority	$\chi^2 = 0.14$, df = 1, $p = 0.71$
Global test	$\chi^2 = 163.0$, df = 5, $p < 0.001$
**Panel B. Time-varying era effect (EpViX vs pre-EpViX)**	
Hazard ratio, 0–12 months	3.97 (3.52–4.46)
Hazard ratio, > 12 months	1.74 (1.57–1.93)

Analysis	Estimate (95% CI)	
Restricted mean time-to-transplant difference (0–60 mo)	≈ 16 months earlier under EpViX	
**Panel C. Absolute probability of transplantation (%)**		
**Time point**	**Adjusted[b] pre / EpViX**	**Unadjusted KM pre / EpViX**
12 months	15 / 34	11 / 36
36 months	31 / 60	30 / 60
60 months	41 / 73	43 / 70
**Panel D. Sensitivity analyses (EpViX era effect)**		
Primary adjusted Cox model	HR 2.55 (2.37–2.74)	
Restricted window (pre 2011–2014 vs EpViX 2016–2019)	HR 2.17 (1.98–2.39)	
Excluding within-era duplicate registrations	HR 2.31 (2.14–2.49)	
Interrupted time-series (level-change at implementation)	IRR 1.89 (1.06–3.36)	

HR, hazard ratio; IRR, incidence rate ratio; KM, Kaplan–Meier; RMST, restricted mean survival time; CI, confidence interval. All Cox models include sensitization (cPRA) and lack-of-access priority status, with robust standard errors clustered at the patient level.

[b] Covariate-standardized (g-formula) absolute probabilities from the Cox model, averaged over the observed covariate distribution and reported under independent censoring (competing events treated as censoring; see Methods). Values are close to the unadjusted Kaplan–Meier estimates, the difference reflecting standardization for the between-era shift in sensitization.

Panel A indicates that the proportional-hazards assumption was not met for the era effect; Panel B therefore reports interval-specific hazard ratios and the restricted mean difference. Panel D shows the era association was preserved across alternative specifications.

Supplementary Table S3. Qualitative review of the 30 false-negative epitope-level virtual crossmatch (eVXM) assays, grouped according to the most likely explanation for discordance with prospective complement-dependent cytotoxicity crossmatch (CDCXM).

Most likely explanation	Assays, n	Predominant CDCXM features*	Transplant proceeded
CDCXM reactivity below the operational MFI threshold used for unacceptable-antigen assignment	15	4/4/4	No
Non-donor-specific antibody reactivity (NDSA)	6	4/4/6	No
Negative solid-phase screening despite CDCXM positivity	4	0/4/6	No
Elevated self-reactivity / high assay background	2	4/4/4	No

No contemporaneous antibody panel available	2	6/8/8	No
Donor-specific antibody reactivity without molecular support from shared eplet analysis	1	4/4/4	No

CDCXM, complement-dependent cytotoxicity crossmatch; AHG, anti-human globulin; DTT, dithiothreitol; IgG, immunoglobulin G. CDCXM characteristics are summarized according to the predominant pattern observed within each explanatory category. Values represent the predominant score pattern across T-cell, B-cell, AHG-augmented, and DTT-treated preparations. Because multiple assays with heterogeneous scores were available for some cases, individual assay-level results are not shown.
